# Recognizing Patterns of Nature Contact Associated with Well-Being: An Exploratory Cluster Analysis

**DOI:** 10.3390/ijerph21060706

**Published:** 2024-05-30

**Authors:** Adriano Bressane, Joao Pedro da Cunha Pinto, Líliam César de Castro Medeiros

**Affiliations:** 1 Institute of Science and Technology, São Paulo State University (UNESP), São José dos Campos 12209-904, Brazil; liliam.medeiros@unesp.br; 2 Graduate Program in Civil and Environmental Engineering, São Paulo State University (UNESP), Bauru 17033-360, Brazil; jp.pinto@unesp.br

**Keywords:** cluster approach, nature contact, mental well-being, multidimensional analysis, developing countries

## Abstract

*Statement of Problem*: Progressive urbanization has reduced human interactions with nature, raising concerns about its impact on mental well-being. Previous research has often focused on specific aspects of nature contact, neglecting its multifaceted dimensions and their effects on mental health, particularly in developing countries. *Research Gap*: There is a scarcity of studies exploring the comprehensive dimensions of nature contact, such as frequency, duration, intensity, and space naturalness, and their correlation with mental well-being in developing countries’ urban settings. *Purpose*: This study aims to identify patterns of nature contact related to mental well-being in metropolitan areas of Brazil using exploratory cluster analysis, bridging the existing knowledge gap and informing targeted interventions to enhance mental health through nature contact. *Method*: An online survey collected data from 2136 participants in Brazil’s metropolitan areas, focusing on their nature interaction patterns and mental health status using the Depression Anxiety and Stress Scale (DASS-21), hierarchical clustering with *p*-values via multiscale bootstrap resampling, and analysis of variance. *Results and Conclusions*: Three distinct groups were identified, showing varied patterns of nature contact and demographic profiles. Greater and more frequent nature contact was associated with lower levels of depression, anxiety, and stress. These findings suggest a beneficial relationship between nature contact and mental well-being. *Practical Implications*: The results underline the importance of urban planning and public health policies that facilitate access to natural spaces, highlighting socioeconomic factors as significant barriers to this access. *Future Directions*: Further research should explore causal relationships and consider the specific realities and challenges faced by residents of developing nations.

## 1. Introduction

Human engagement with nature has emerged as a subject of increasing interest within academic research, particularly regarding its impact on mental well-being. The progressive urbanization of our daily environments has led to a diminished frequency of interactions with green and natural spaces. This development underscores the critical need for research into how varied patterns of nature contact may influence individuals’ mental health, as highlighted by Ningtyas et al. [1], who explored the restorative effects of nature exposure on stress, anxiety, and depression levels in Indonesia, emphasizing its role in enhancing emotional well-being. The justice in access to urban nature is central to this discourse, as explored by Langhans et al. [2], who emphasize the necessity for equitable access to green spaces to improve mental well-being across different communities.

Global changes and the advancement of urbanization contribute to a growing disconnection between humans and the natural environment. This separation poses significant implications for mental health, especially given the expanding body of evidence indicating that nature contact supports psychological well-being. Fornara et al. [3] have shown how contact with nature can significantly reduce anxiety during times of social deprivation, such as the COVID-19 pandemic, highlighting the resilient role of nature in urban settings. 

Although the significance of nature contact is widely acknowledged, previous research has often focused on specified aspects of this interaction. There remains a scarcity of studies exploring the multifaceted dimensions of nature contact, aiming to identify patterns that yield mental health benefits. Moreover, in developing countries such as Brazil, social and economic inequalities exacerbate challenges, potentially restricting access to quality green spaces. Talal and Gruntman [4] examination of changes in visiting urban nature sites during the COVID-19 pandemic in Israel further illustrates the dynamic nature of human–nature interactions in urban settings.

Prior studies have provided valuable insights into the general benefits of nature contact for mental health. Nonetheless, there is a lack of integrated, multidimensional analyses concerning the patterns of nature contact (e.g., frequency, duration, intensity, and the natural quality of the space) and their correlation with mental well-being in the urban settings of developing countries. 

Pasanen et al. [5] discuss the importance of urban green spaces for individuals living alone, focusing on the mediating roles of relational and collective restoration, which are crucial for mental health in densely populated areas. Furthermore, the majority of studies have focused on populations in developed countries, with limited exploration into the specific realities and challenges faced by residents of developing nations. This knowledge gap highlights the necessity for exploratory studies that examine the multidimensional aspects of nature contact and its relationship with mental health across diverse contexts. For instance, Xie et al. [6] underscore the importance of activities like bird watching for strengthening residents’ psychological well-being and fostering crucial human–nature connections within living spaces in Beijing, which resonates with the need for such multidimensional exploration. Moreover, the interaction with nature is not limited to outdoor environments. Ma [7] highlights the psychological impacts of caring for houseplants on mental well-being and mindfulness in Chinese adults, suggesting that even indoor nature contact can serve as a vital component of mental health strategies.

This study aims to explore patterns of nature contact related to mental well-being in metropolitan areas of Brazil through exploratory cluster analysis. We intend to identify distinct groups based on their nature contact patterns and assess how these patterns correlate with mental well-being indicators, such as levels of depression, anxiety, and stress. By doing so, we aim to bridge the existing knowledge gap and offer insights on how targeted interventions can be designed to enhance mental well-being through nature contact in urban settings. The anticipated outcomes of this study are expected to contribute to the environmental health and psychological well-being knowledge base, emphasizing the importance of equitable access to quality natural spaces as a matter of public health and social justice, echoing Ignatieva et al. [8] regarding the need to understand the specific socio-political, historical, cultural, and ecological contexts when interpreting urban natures and designing nature-based solutions. The neurobiological effects of urban built and natural environments on mental health, systematically reviewed by Bolouki [9], also echo the need for the multidimensional exploration of nature contact and its benefits for mental well-being.

## 2. Materials and Methods

A comprehensive online survey collected data from participants in Brazil’s metropolitan areas, adhering to the nation’s ethical standards for human research (Ethical Approval #58149622.3.0000.0077). Of the approximately 10,000 residents approached, 2136 completed the survey. 

Recruitment strategies combined online and in-person methods to secure a representative sample across a broad demographic spectrum. The primary method involved digital platforms, utilizing targeted social media campaigns and emails to educational institutions to reach diverse audiences. This was supplemented by strategic partnerships with local organizations, such as public libraries, to include underrepresented groups, thereby enhancing sample diversity. A stratified random sampling technique was employed to capture varied socioeconomic and demographic profiles through both online engagement, via social media and forums, and direct outreach in community spaces like parks and centers. Periodic reminders targeted less represented groups to ensure inclusivity and maximize participation. This cohesive, multifaceted approach was crucial for assembling a diverse cohort, ensuring the robustness and generalizability of the findings.

The questionnaire was structured into distinct segments to evaluate the participants’ interaction with nature and their psychological well-being (Table 1). Initially, it gathered data on how frequently the participants engage with nature each week, providing response options like “<1 time”, “1 time”, “2 times”, “3 times”, or “>3 times”. 

The survey then explored the length of these engagements with nature on a weekly basis, offering a selection range from “30 min” to “over 240 min”, increasing by 30 min intervals. It also inquired about the types of activities participants undertook during their time in nature, later categorizing these activities by their intensity: low, moderate, or high. Subsequently, the questionnaire asked about the specific locations of these nature interactions over the last week, with areas near more untamed environments rated as having higher naturalness, and others considered to have lower naturalness. Additionally, this study also inquired about the accessibility and suitability of natural areas for visitation, leisure, and recreational activities, whether available near the respondents’ residences or not.

For mental well-being assessment, the survey’s final segment included the Depression, Anxiety, and Stress Scale (DASS-21), a reputable and validated measure for psychological evaluation [10]. The DASS-21 employs a four-point Likert-type scale for its responses, where ‘0’ signifies “never”, ‘1’ represents “occasionally”, ‘2’ is “quite often”, and ‘3’ indicates “most of the time”. 

This scale helps in assessing symptoms associated with depression, anxiety, and stress. Questions within this scale, for example, address issues such as difficulty relaxing, indicative of stress; physiological signs of anxiety like dry mouth and shortness of breath not due to physical exertion; and symptoms of depression, including a lack of positive emotions and feelings of life being meaningless (Table 2).

Initially, a two-way analysis of variance [11] was employed to examine the interactions between demographic factors (including gender, age, income, and education level) and patterns of contact with nature (characterized by the naturalness of the space, intensity, frequency, and duration of nature interactions) to identify their potential effects on mental well-being indicators (specifically depression, anxiety, and stress levels). Subsequently, a cluster analysis using ‘pvclust algorithm’, a hierarchical clustering with *p*-values via multiscale bootstrap resampling [12], was employed to investigate hypothetical associations between patterns of nature contact and mental well-being. 

For the present study, we consider nature contact to be direct engagement with outdoor natural environments, which includes activities such as walking in nature, participating in recreational activities in green spaces, and other forms of active interaction within urban and peri-urban natural settings. Furthermore, our study encompasses a broad definition of “nature” that includes various types of environments where individuals can experience natural elements. This includes not only urban wilderness and maintained parks but also agricultural lands and spaces with indoor greening. Each of these environments offers different degrees of natural exposure and has been considered to evaluate the diversity of nature contact patterns.

As the two-way ANOVA revealed no statistically significant interactions (*p* > 0.05), cluster analysis was undertaken without performing sensitivity tests to determine whether associations significantly varied across different subgroups (e.g., age, gender). With the establishment of the clusters, subsequent analysis sought to verify distinct patterns, which were statistically compared via Welch’s ANOVA and Games–Howell post hoc tests [13], aiming to elucidate significant differences in mental well-being associated with contact with green areas. 

Effect sizes were quantified utilizing Cohen’s d [14]. All statistical analyses were conducted with a test power of 0.8 and a significance level (α) of 0.05, targeting a minimum detectable effect size (ρ) of 6%. A power test (1 − β) of 0.8 ensured an 80% chance to detect significant effects, with a significance level (α) of 0.05 and a minimum detectable effect size (rho) of 5%, minimizing Type II errors and controlling the false discovery rate [15].

## 3. Results

### 3.1. Cluster Overview

The demographic composition of the 2136 respondents is as follows: 59.6% were female. The age distribution included 15.7% young adults aged 18–25 years, 55.2% adults aged 26–45 years, and 29.1% middle-aged adults aged 46–65 years. Regarding income, respondents were categorized into four groups: 16.2% earned up to two minimum wages (low income), 21.9% earned between two and four minimum wages (lower-middle income), 37% earned between four and ten minimum wages (middle income), and 17.7% earned between ten and twenty minimum wages (upper-middle income). Educational attainment among the respondents was high, with 83.5% holding a university degree, 16.2% having completed high school, and only 0.3% possessing elementary education only.

**Figure 1:**
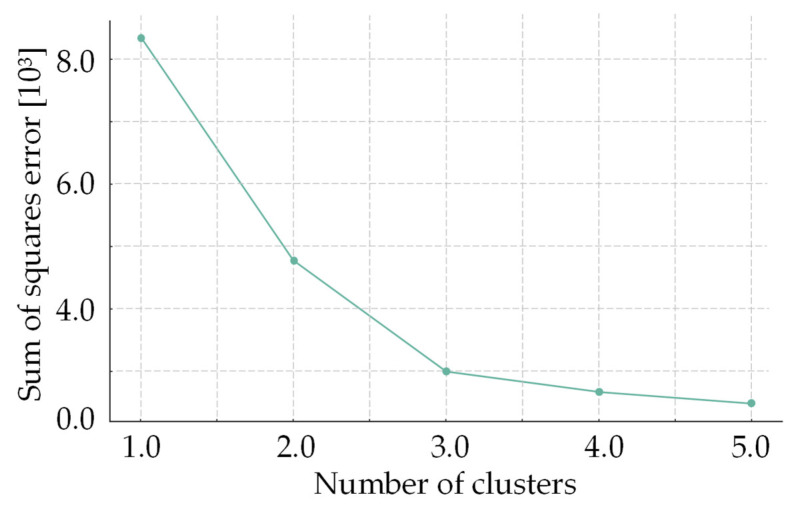
Balance between number of clusters and reduction of errors.

The cluster analysis revealed the existence of three significantly distinct groups (*p* < 0.05), reflecting different patterns of contact with nature and varied demographic profiles. The graph in Figure 1 illustrates how the sum of squares error (SSE) decreases as we increase the number of clusters. Considering the balance between the number of clusters and the explained variance, three corresponded to the optimal number of clusters, equivalent to the point where the reduction in SSE begins to diminish more slowly.

**Figure 2:**
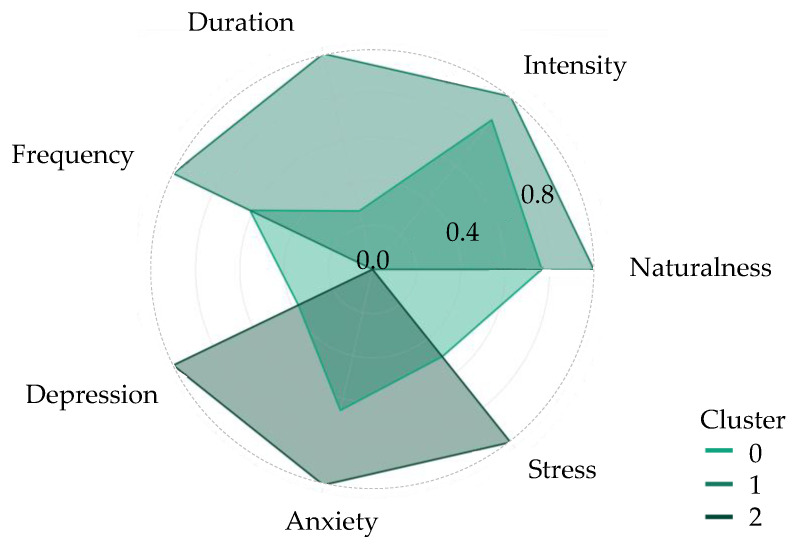
Variations in patterns of nature contact and mental health indicators.

Figure 2 illustrates variations in nature contact patterns and mental health indicators. A visual analysis of these variations suggests that Cluster 0 may represent individuals with a tendency towards greater access to and engagement with natural areas, possibly reflecting a more active lifestyle or a higher appreciation for time spent outdoors. This cluster, comprising 58.4% of the study population, includes individuals who have a higher frequency, duration, and intensity of contact with nature. 

In contrast, Cluster 1, which accounts for 26.0% of participants, predominantly includes individuals with less nature contact. The findings point towards a more urban lifestyle with limited access to green spaces for this cluster, highlighting the importance of urban planning in mitigating these disparities. 

Cluster 2 exhibits intermediate characteristics of nature contact, representing 15.6% of the sample, with members displaying a balanced interaction pattern that differs significantly from the more extreme clusters. This cluster includes a youthful demographic, predominantly younger individuals, who account for 72.4% of its composition, reflecting a unique blend of engagement levels with nature. Members of Cluster 2 are characterized by their educational background, with 100% having completed only high school education, and face significant economic challenges, with 44.5% belonging to the low-income tier. This socioeconomic backdrop shapes their access to and interaction with natural environments, contributing to a diverse range of nature contact experiences that include both moderate and occasional high-intensity activities. The infrastructure reported by this group is mixed, with 39.0% citing lack of adequate green space, yet 36.2% finding the existing infrastructure sufficient. Their preference for naturalness is equally varied, with 51.7% favoring wilderness areas while 35.9% prefer urban green spaces, indicating a flexible approach to engaging with nature. This cluster’s pattern of engagement—reflected in the time they spend in nature and the frequency of their visits—suggests a more integrated approach to urban and natural environments, pointing to the potential for targeted public health interventions to enhance their access and engagement with nature.

Cluster 2 displays varied levels of engagement with natural spaces, which directly influences their mental health outcomes. Members of this cluster experience moderate levels of depression, anxiety, and stress compared to the other clusters. This is particularly notable as these individuals have access to both urban and natural environments but face certain barriers that limit their full engagement with nature. The evidence suggests that while there is some benefit from nature contact in reducing mental distress, the extent of these benefits is less pronounced in Cluster 2 due to their less frequent and intense interactions with nature. This relationship highlights the need for targeted interventions that could enhance the quality and frequency of nature interactions for this cluster to optimize mental health benefits.

### 3.2. Gender and Age Composition

The analysis of the distribution by gender and age composition in the clusters provides essential insights into the dynamics of interaction with urban green spaces. In Cluster 0, there is a gender-balanced distribution (54.1% female and 45.9% male) and a predominance of adults (59.7%), complemented by a significant proportion of middle-aged people (25.5%) and seniors (10.2%). Since Cluster 0 groups individuals with greater access to and involvement with natural areas, it is inferred that such an audience is mature, showing interests in outdoor activities that are shared by both genders. 

In contrast, Cluster 1 is characterized by a significant female predominance (70.3%), along with an age composition skewed towards smaller proportions of middle-aged individuals (19.6%) and youth (6.7%). This configuration suggests a particular attraction of women to this cluster, possibly due to a lower interaction with the natural environment, highlighting the need for inclusive strategies to increase engagement with nature, especially among the younger and female audience. In Cluster 2, the continuation of female predominance is observed (with a relatively balanced male participation of 35.9%) alongside a notable preponderance of young people (72.4%), suggesting an intermediate level of engagement of these individuals with green spaces. This setup reveals a unique dynamic, with a minority of adults (19.2%) and middle-aged people (6.2%), emphasizing the importance of adapting engagement strategies to meet the needs of a younger audience.

### 3.3. Education Level and Income

Insights into the educational background and economic tiers of cluster members elucidate the patterns of engagement with urban green areas, affected by both educational and economic considerations. In Cluster 0, the predominance of members with university education (99.3%) aligns with a majority middle-income class (40.2%), followed by lower-middle (19.2%) and upper-middle (22.2%) income classes. This profile suggests a high level of awareness about the importance of green spaces and facilitated access to them, potentially due to a more stable economic capacity that supports frequent and meaningful interaction with nature. 

For Cluster 1, the near totality of members with university education (98.2%) contrasts with a higher presence of low-income individuals (16.0%) compared to Cluster 0. This combination indicates that, despite a high level of human capital, economic issues may negatively influence the availability and engagement with quality green spaces, being reflected in infrequent or short-duration interactions with nature. In Cluster 2, the distinct reality of 100% of members having only high school education is markedly associated with a high concentration of individuals in the low-income class (44.5%). This configuration highlights significant barriers to access to and quality of green spaces compared to Cluster 0, suggesting that educational and economic limitations combine to profoundly affect attitudes, behaviors, and opportunities for interaction with urban natural environments.

### 3.4. Infrastructure and Naturalness

The synthesis of views on the infrastructure of green areas and the desired level of their naturalness offers key insights into understanding human interactions in urban landscapes. For Cluster 0, the majority of individuals report the existence of adequate infrastructure (49.2%), which aligns with a significant preference (78.8%) for areas of high naturalness. This scenario indicates a valuation of green spaces that resemble natural ecosystems, suggesting that the members of this cluster benefit from functional green space infrastructure conducive to wilder, less anthropogenic experiences, potentially due to effective urban policies prioritizing the maintenance and creation of accessible and natural green areas. 

In Cluster 1, the perception of green space infrastructure as non-existent (40.9%) or inadequate (27.0%) is prevalent, reflecting challenges in the availability and quality of these spaces. Simultaneously, the members’ preference for built urban spaces with some degree of naturalness (54.6%) and a low inclination towards wilderness areas (6.7%) highlight a demand for the harmonious integration of natural elements into built environments, emphasizing the need for improvements in existing infrastructure and the development of new spaces that balance nature and urbanization. 

Cluster 2 presents a mixed situation, with balanced reports between the lack of adequate infrastructure (39.0%) and its adequacy (36.2%), as well as a more balanced distribution of preferences for wilderness (51.7%) and built urban areas with green spaces (35.9%). This suggests a diversity of expectations and an opportunity for urban planning approaches that consider both the preservation of wilderness areas and the integration of green spaces into urban environments, aiming to meet a broader spectrum of preferences and needs of this cluster’s members.

### 3.5. Duration and Frequency

The results concerning time spent in nature and interaction frequency with nature by individuals in each cluster give a more detailed insight into their engagement with natural spaces. For Cluster 0, the combination of a higher proportion of individuals spending more than 240 min in nature per week (24.0%) with a relatively high frequency of contact (30.3% “oftentimes” and 28.1% “sometimes”) underscores a lifestyle that significantly values nature and integrates it into daily activities. 

This profile reflects a notable commitment to outdoor time, whether for leisure or exercise, emphasizing the importance of green spaces to this group. In contrast, Cluster 1 presents a striking difference, with the majority of individuals spending 1 to 30 min (52.8%) and 31 to 60 min (31.0%) per week in nature, coupled with a high percentage (49.9%) that almost never engages with natural environments. This pattern suggests significant limitations in available time or accessibility to green spaces, indicating the need for public policies to encourage contact with natural environments and overcome barriers to access. For Cluster 2, the more evenly distributed time spent in nature, with an emphasis on the 31 to 60 min range (27.1%), and a diversity in contact frequency (29.1% “sometimes” and 17.8% “oftentimes”) reflects a balance in nature interactions. The lower proportion of “almost never” (15.3%) compared to Cluster 1 points to a more regular integration of nature into this group’s routines.

### 3.6. Intensity of Engagement

The distribution of intensity level in nature engagement offers a distinct perspective on the behavioral predispositions of each cluster. In Cluster 0, nearly half of the individuals (47.1%) engage in high-intensity activities, such as trail running, while a similar portion (44.9%) prefer moderate activities. The relatively low proportion of low-intensity interactions (6.8%) and the minimal portion that does not participate in such activities (1.2%) suggest a tendency of this group to favor active lifestyles, where vigorous interaction with nature is a significant dimension. 

Conversely, Cluster 1 is characterized by a predominance of members who do not engage in physical activities in nature (53.2%), indicating a less active stance or possible barriers, whether due to personal preferences, physical limitations, or the absence of suitable infrastructure. Only a minority (2.9%) engages in intense activities, contrasting with a greater inclination towards low-intensity (20.8%) or moderate (23.1%) activities, which may indicate a preference for more passive or reflective interactions with nature. 

Cluster 2 presents an intermediate profile, with 30.5% of individuals engaged in intense activities and 36.8% in moderate ones, aligning with a pattern of active interaction, yet less extreme than Cluster 0. The considerable level of non-participation (20.4%) also draws attention, indicating that a significant portion of respondents in this cluster lack access to or interest in contact with natural spaces.

### 3.7. Association with Mental Well-Being

**Figure 3:**
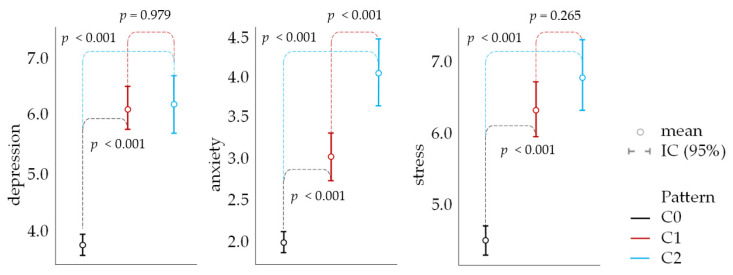
Relationship between patterns of nature contact and mental distress.

The evidence detailed in Table 3 and illustrated in Figure 3 supports the existence of a positive correlation between engagement with natural environments and mental health outcomes. These findings suggest that individuals who experience more frequent and prolonged exposure to natural settings are likely to report reduced symptoms of depression, anxiety, and stress. This association underscores the therapeutic potential of nature, emphasizing that access to and the quality of green spaces can play a critical role in enhancing mental well-being. The analysis goes further to imply that the characteristics of these natural spaces—such as their level of naturalness, infrastructure, and the types of activities they support—may significantly influence the extent of these mental health benefits.

This expanded understanding provides a compelling case for the prioritization of green space development as a public health intervention, aiming to create environments that support mental health and foster a deep, beneficial connection with nature. However, it is important to remember that these are exploratory findings suggesting associations that do not establish causality. Table 4 synthesizes the diverse and complex relationships among the three clusters regarding their engagement with natural environments.

## 4. Discussion

This study reinforces the critical importance of nature contact for mental well-being, a thesis consistently corroborated by the recent academic literature. Grounded in the previous work of Ningtyas et al. [1], Fornara et al. [3], James et al. [16], and Costa et al. [17], and complemented by Özgüner (2019), this research not only corroborates the restorative effects of engaging with natural environments on mitigating symptoms of stress, anxiety, and depression but also significantly extends these insights. By delving into the nuanced relationship between diverse patterns of nature contact and mental health outcomes, this study unveils the complex dynamics underlying this interaction, suggesting that the psychological benefits derived from nature are not universally consistent across different demographics but are influenced by the intensity, frequency, and type of nature engagement.

In alignment with our findings, Ningtyas et al. [1] reveal how exposure to nature can reduce stress, anxiety, and depression, underscoring the necessity of incorporating natural spaces into urban planning, particularly in areas like West Bandung Regency, Indonesia, to foster mental well-being. James et al. [16] and Costa et al. [17] emphasize the importance of integrating natural spaces into urban contexts for cognitive restoration and mental well-being enhancement, corroborating the urgency to promote environmental justice and public well-being in cities.

This study advances by identifying specific patterns of nature contact, directly associating them with mental well-being indicators, enriching and expanding the pre-existing theory relating to the psychological benefits of nature, as discussed by Costa et al. [17] and Özgüner [18]. The identification of three clusters, each presenting distinct patterns of nature interaction and differentiated demographic profiles, challenges the widespread notion that all forms of nature contact are uniformly beneficial, illustrating the complexity of this relationship.

Highlighting the findings from recent investigations by Talal and Gruntman [4] and Xie et al. [6], this work adopts an integrated analysis that appreciates the multifaceted nature of human interaction with natural environments. This approach is pivotal, demonstrating that the quality and form of nature contact play crucial roles in influencing mental health benefits, a perspective further supported by Spano et al. [19] and Richardson et al. [20]. These scholars stress the importance of considering these aspects in urban and public health policy planning to ensure environmental justice and the development of accessible green spaces.

The research presented here identifies three distinct clusters of nature interaction, each with unique demographic and behavioral characteristics, challenging the notion that all forms of nature contact are uniformly beneficial. This differentiation not only enriches existing theories on the psychological benefits of nature contact but also provides a deeper understanding essential for maximizing mental health benefits. For instance, Barnes et al. [21], Oh et al. [22], and Reuben and Himschoot [23] explore how different elements of nature and participatory experiences contribute variably to mental health improvements, depending on the individual’s intensity of connection with nature.

Furthermore, this study shines a light on the socioeconomic barriers that significantly impact access to natural spaces, revealing profound disparities among social groups. It underscores that individuals in Cluster 0, characterized by more intense and quality nature contact, often possess higher incomes and educational levels, thus enjoying superior access to natural spaces. In stark contrast, Clusters 1 and 2 exhibit reduced nature contact and quality of interaction, highlighting socioeconomic and urbanistic challenges that particularly afflict lower social class youth. These insights are echoed in recent research, such as Robinson et al. [24], who identify psychosocial and economic barriers impacting racialized individuals’ access to green spaces, and Ibes, Rakow, and Kim [25], who explore the specific barriers preventing youth of color from engaging with nature.

These insights underscore the urgency for public policies and urban interventions aimed at enabling equitable access to natural spaces, which is further substantiated by Noël et al. [26], who discuss social obstacles in the utilization of public green spaces, and Groulx, Freeman, and Lemieux [27], who review the accessibility of nature in areas beyond urban boundaries. Edwards, Larson, and Burdsey [28] highlight the particular barriers faced by Muslim communities in the UK, suggesting that strategies to enhance both access and the quality of these spaces must consider these multifaceted barriers to ensure equitable access across all demographics.

This alignment with global urban planning and public health policy objectives advocates for interventions that can substantially benefit mental well-being, especially in metropolitan regions of developing countries, like Brazil. The exploration of nature’s intricate role in promoting mental well-being herein establishes a strong foundation for further studies and policy formulations. Despite acknowledging limitations such as its exploratory nature and focus on metropolitan areas in Brazil, this study is a significant stride towards harnessing nature’s therapeutic potential to enhance public health and well-being worldwide. Through a robust analysis and the synthesis of findings from pioneering researchers, this work contributes to the ongoing discourse on environmental justice and mental health, offering compelling evidence for the creation of green spaces accessible to all, irrespective of socioeconomic status.

## 5. Recommendations for Green Space Planning for Enhanced Mental Well-Being

### 5.1. Diverse Natural Spaces for Varied Community Needs

Our study’s findings underline the importance of diversifying the types of green spaces within urban environments to cater to different community preferences and needs, as evidenced by the distinct clusters identified. For example, Cluster 0 benefitted significantly from active engagement in robust natural settings, suggesting that areas with substantial natural features and opportunities for intense physical activities should be developed. Research highlights the link between vegetation complexity and diverse microbial communities in urban green spaces, which can influence human health outcomes [29].

### 5.2. Inclusive Design for Enhanced Accessibility

The demographic analysis of Cluster 2, characterized by younger and economically disadvantaged individuals, highlights the need for inclusive design in urban planning. Green spaces should be accessible in terms of both physical accessibility and socioeconomic factors, such as free or low-cost entry. Studies on community public open space planning based on green infrastructure prioritize biodiversity, which can also address the needs of diverse community groups [30].

### 5.3. Integration of Natural Spaces into Urban Fabric

For Cluster 1, which showed limited interaction with nature and a tendency towards an urban lifestyle, integrating smaller green patches and biophilic design elements into the urban fabric can enhance daily nature contact. Research underscores the importance of diverse green infrastructure in improving the well-being of urban communities through varied experiential contacts [31].

### 5.4. Robust Infrastructure for Sustainable Engagement

The findings suggest that proper infrastructure in green spaces, as preferred by Cluster 0, significantly enhances the quality of engagement and its psychological benefits. Research on the revitalization of green open spaces has shown that proper planning and infrastructure development can fulfill the varied needs of urban communities, enhancing engagement and reducing pollution [32].

### 5.5. Policy Framework and Community Involvement

Finally, our study advocates for a policy framework that supports the sustainable development of urban green spaces, coupled with active community involvement in the planning process. This ensures that the spaces meet the actual needs of the community and are maintained effectively over time. Evidence from studies on bird diversity in urban green spaces supports the integration of ecological considerations in urban planning to maintain biodiversity and community well-being [33].

## 6. Final Remarks

This study sought to fill an existing knowledge gap by exploring the multifaceted dimensions of nature contact—frequency, duration, intensity, and naturalness of space—and their correlation with mental well-being in urban contexts of developing countries, with a special focus on metropolitan areas in Brazil. Utilizing exploratory cluster analysis, this study identified distinct patterns of nature contact that correlate with mental health indicators, providing a basis for targeted interventions that enhance mental well-being through engagement with nature.

Cluster analysis revealed three significantly distinct groups, reflecting varied patterns of nature contact and demographic profiles. The findings clearly demonstrate a beneficial relationship between more intense and frequent nature contact and lower levels of depression, anxiety, and stress. These results underline the complexity of nature–human interactions and suggest that the psychological benefits derived from nature contact are not universally consistent, but vary according to different demographics and patterns of nature contact.

This research study significantly advances the understanding of the relationship between nature contact and mental well-being, offering a nuanced analysis that surpasses previous studies. It contributes to knowledge in environmental health and psychological well-being by demonstrating how specific patterns of interaction with nature correlate with mental health benefits. This study enriches existing theories on the psychological advantages of nature contact and provides a deeper understanding essential for maximizing mental health benefits through personalized public health and urban planning interventions.

The results emphasize the importance of urban planning and public health policies that facilitate access to natural spaces, especially considering socioeconomic factors as barriers. This study underlines the need for public policies and urban interventions aimed at guaranteeing equitable access to natural spaces, advocating for the development of green spaces accessible to all, regardless of socioeconomic status.

Future research should focus on exploring causal relationships between nature contact and mental well-being and consider the specific realities and challenges faced by residents of developing nations. Investigating the impact of different types of nature-based interventions on diverse population groups can provide further insights into how to effectively enhance mental well-being through nature contact. Our study did not specifically analyze the minimum sizes of green spaces required to influence mental health benefits, nor did it address the differential impacts of solitary versus crowded nature experiences. Nevertheless, we acknowledge the significance of this aspect and its implications for urban planning and mental health. Therefore, we highlighted the need for future research to specifically investigate how different social settings within natural environments influence mental well-being, as well as how novel studies could focus on defining specific size thresholds that optimize mental health outcomes.

In conclusion, the distinctions noted among the clusters in our study do indeed present a compelling case for the application of urban planning principles that align with the specific needs and characteristics of each cluster. In this sense, we recognize the potential for future research to delve deeper into these distinctions to inform more tailored urban interventions. Such an approach could involve the development of specialized green space projects or nature-based solutions that are specifically designed to address the unique environmental interactions and mental health outcomes identified within each cluster. This could indeed enhance the effectiveness of urban planning and public health policies, ensuring that they are not only comprehensive but also contextually relevant to the different segments of the urban population.

## Figures and Tables

**Table 1:** Questionnaire overview with same examples of key questions.

Question Examples	Options
What is your gender?	( ) male ( ) female ( ) non-binary ( ) other (please specify): _______
What is your age range?	( ) 18–24 years ( ) 25–45 years ( ) 46–60 years ( ) 61 years or older
What is your highest level of completed education?	( ) elementary education ( ) high school ( ) university degree
Which natural locations did you visit in the last seven days?	locations: (please specify): _______
Which physical activities did you engage in while in contact with nature in the last seven days?	activities: (please specify): _______
How often do you visit the urban green spaces in your neighborhood?	( ) <1/week ( ) 1/week ( ) 2/week ( ) 3/week ( ) >3/week
How long do you usually spend in urban green spaces during each visit (minutes)?	( ) <30 ( ) 31–60 ( ) 61–90 ( ) 91–120 ( ) 121–150 ( ) 151–180 ( ) 181–210 ( ) 211–240 ( ) >240
How would you rate the suitability of the UGS in your neighborhood for visits and recreation?	( ) none ( ) unsuitable ( ) suitable

**Table 2:** DASS-21 questionnaire overview.

Item	Dimension	Question Description	Response *
1	Stress	I found it hard to wind down.	0–3
2	Anxiety	I was aware of dryness of my mouth.	0–3
3	Depression	I couldn’t seem to experience any positive feeling at all.	0–3
4	Anxiety	I experienced breathing difficulty	0–3
5	Depression	I found it difficult to work up the initiative to do things.	0–3
6	Stress	I tended to over-react to situations.	0–3
7	Anxiety	I experienced trembling (e.g., in the hands).	0–3
…	…	…	…
21	Depression	I felt that life was meaningless.	0–3

* Note: Scale for all items ranges from 0 (Did not apply to me at all) to 3 (Most of the time).

**Table 3:** Effect of patterns of nature contact on mental distress.

			Cluster 1	Cluster 2
depression	cluster 0	difference	−2.32 ***	−2.38 ***
		$d_{Cohen}$	0.62	0.63
	cluster 1	difference	-	−0.06
		$d_{Cohen}$	-	*ns*
anxiety	cluster 0	difference	−1.05 ***	−2.10 ***
		$d_{Cohen}$	0.36	0.71
	cluster 1	difference	-	−1.05 ***
		$d_{Cohen}$	-	0.38
stress	cluster 0	difference	−1.84 ***	−2.34 ***
		$d_{Cohen}$	0.45	0.58
	cluster 1	difference	-	−0.49
		$d_{Cohen}$	-	*ns*

*** *p* < 0.001; *ns* = non-significant effect.

**Table 4:** Characteristics and diversity of clusters in nature contact patterns.

Attribute	Cluster 0 (High Engagement)	Cluster 1 (Limited Engagement)	Cluster 2 (Moderate Engagement)
Population	58.4%	26.0%	15.6%
Gender	Balanced (54.1% female, 45.9% male)	Female predominance (70.3%)	Female predominance (35.9% male)
Age	Adult predominance (59.7% adults, 25.5% middle-aged, 10.2% seniors)	Younger skew (6.7% youth, 19.6% middle-aged)	Youth predominance (72.4% youth, 19.2% adults)
Income	Mostly middle income (40.2% middle, 22.2% upper-middle)	High education, lower income (16.0% low-income)	High concentration in low-income class (44.5%)
Education	High (99.3% university-educated)	High (98.2% university-educated)	Lower (100% high school education)
Natural Access	High frequency, duration, and intensity of nature contact	Limited access and interaction	Moderate access and interaction, diverse preferences
Infrastructure	Adequate (49.2% report adequate infrastructure)	Mostly inadequate or non-existent (67.9% combined)	Mixed reports on infrastructure adequacy (balanced)
Preference	High (78.8% prefer high naturalness)	Mixed, leans towards built environments (54.6%)	Balanced between wilderness and UGS (51.7% wilderness)
Time Spent in Nature	Over 240 min per week (24.0%)	1 to 60 min per week (majority)	31 to 60 min range, more evenly distributed
Intensity of Engagement	High-intensity activities (47.1%)	Low engagement (53.2% do not engage)	Moderate to intense activities (30.5% intense, 36.8% moderate)
Health Outcomes	Positive association with reduced mental distress	Increased distress due to limited nature interaction	Moderate benefits, varied based on the level of engagement

## Data Availability

Ethical restrictions: due to the nature of this research, participants in this study did not agree for their data to be shared publicly, so supporting data are not available.
